# Editorial: Understanding malicious behaviors on digital platforms

**DOI:** 10.3389/fpsyg.2024.1384226

**Published:** 2024-03-04

**Authors:** Seongcheol Kim, Dam Hee Kim, Changi Nam, Ahran Park

**Affiliations:** ^1^School of Media and Communication, Korea University, Seoul, Republic of Korea; ^2^School of Business and Technology Management, Korea Advanced Institute of Science and Technology, Daejeon, Republic of Korea

**Keywords:** platforms, malicious behaviors, psychological understanding, malicious comments, hate speech, cyberbullying

## Introduction

The value of digital platforms cannot be ignored. With their integrated nature, digital platforms remove boundaries in the digital economy and have become the operating system of our lives (Vaidhyanathan, 2018, p. 99). Digital platforms are not an option anymore but rather an essential tool and the core of the digital ecosystem (Ha et al., 2023). In the meantime, the capability to utilize digital platforms determines not only opportunities but also threats; accordingly, the use of digital platforms has positive and negative consequences. For example, people can take part in open discussions with others on digital news platforms. However, the anonymity and remoteness of digital platforms may allow antisocial behaviors such as the mass production of rumors and public opinion manipulation.

Though the use of digital platforms has both sides of the coin, research on psychological understanding of malicious behaviors on digital platforms is still limited. Previous studies appear to focus mainly on the positive side of the coin. Therefore, this Research Topic solicited empirical articles examining the antecedents, processes, and effects of malicious behaviors on digital platforms. This editorial piece aims to provide a quick review of the four articles published under this Research Topic, followed by concluding remarks.

## Contributions

The four articles take a deep dive into three prevalent forms of malicious behaviors on digital platforms: malicious comments, hate speech, and cyberbullying. First, focusing on malicious news comments, Lee et al. investigate individual factors, including demographic characteristics, personality traits, and reading-related factors, as well as contextual factors such as issue involvement, perceived peer behavior, and the presence of malicious comments in news articles. An analysis of online survey data of 1,000 Koreans demonstrates that most of the proposed variables have a significant impact on the perceived maliciousness of online news comments, except for morality and issue involvement. The results shed light on the mechanisms behind individuals' perception of the maliciousness of online news comments and offer valuable insights into the ways to reduce malicious comments.

Second, two studies tackle hate speech, both its expression patterns in the context of gerontophobia and the public's attitudes toward its regulation. Kim and Ryu have analyzed 133,218 news articles about the elderly and 1,238,935 comments on Naver, Korea's leading portal site, between May 2017 and June 2023. Kim and Ryu have used a deep learning model, kcBert, for labeling and classification of gerontophobic comments, and LDA (Latent Dirichlet Allocation) Topic Modeling for identification of news topics. Over the observed 6 years, the proportion of gerontophobic comments, particularly those showing the “fear of aging,” has gradually decreased. Gerontophobic comments tend to emerge under news articles related to the COVID-19 pandemic, the issues related to the elderly (e.g., their digital and financial exclusion, their economic and social welfare), and other historical issues (e.g., comfort women).

Park et al. unpack factors that predict the public's support for regulation on online hate speech. Through an analysis of online survey data of 1,000 Koreans, Park et al. document two direct pathways to support for regulation from victimization experiences by hate speech and effectiveness of regulatory measures, respectively. Their results also identify an indirect pathway linking (i) content uploading behavior, (ii) victimization experiences by hate speech, (iii) social harm caused by hate speech, and finally, (iv) support for regulation. Park et al. highlight the important roles of perceived harm by hate speech and effectiveness of regulatory measures in determining support for regulation of online hate speech.

Lastly, Al-Turif and Al-Sanad investigate digital bullying, specifically its prevalent forms, causes, and repercussions. Through a descriptive analysis of survey data of 640 students from five universities randomly selected to represent five regions of Saudi Arabia, Al-Turif and Al-Sanad show that digital bullying is widespread in diverse forms on social media (e.g., hostile messages that hurt the feelings of the recipient). For perceived causes of digital bullying, respondents have selected psychological reasons, followed by social, technological development-related, and economic reasons. The results also demonstrate that digital bullying has serious repercussions for social media users, families of victims, and society.

## Conclusion

In summary, the articles provide timely findings and point to the importance of understanding psychological characteristics of malicious behaviors on digital platforms. They advance our understanding of malicious behaviors on digital platforms by showcasing their patterns, causes and effects and delving into mechanisms behind individuals' perceptions of maliciousness as well as support for regulation. Insights gained from this Research Topic could help us better understand the related studies conducted in Asian and Middle Eastern contexts. We hope that this Research Topic will inspire further in-depth research on how to mitigate the serious problems of malicious comments, hate speech and digital bullying on digital platforms.

## Author contributions

SK: Conceptualization, Funding acquisition, Supervision, Writing—original draft, Writing—review & editing. DK: Validation, Writing—original draft, Writing—review & editing. CN: Validation, Writing—review & editing. AP: Validation, Writing—review & editing.
